# Case report: Novel frameshift mutation in *LAMA2* gene causing congenital muscular dystrophy type 1A

**DOI:** 10.3389/fgene.2023.1158350

**Published:** 2023-06-20

**Authors:** Natalia Diaz-Lombana, Lorena Diaz-Ordoñez, Juan David Gutierrez-Medina, Harry Pachajoa

**Affiliations:** ^1^ Centro de Investigaciones en Anomalías Congénitas y Enfermedades Raras (CIACER), Universidad Icesi, Cali, Colombia; ^2^ Departamento de Ciencias Básicas Médicas, Facultad de Salud, Universidad Icesi, Cali, Colombia; ^3^Centro de Investigaciones Clínicas, Fundación Valle del Lili, Cali, Colombia; ^4^Genetic Division, Fundación Valle del Lili, Cali, Colombia

**Keywords:** case report, congenital muscular dystrophy type 1A, laminin subunit alpha 2 *LAMA2*, rare diseases, mutation, exome sequencing, sequence analysis

## Abstract

Congenital muscular dystrophy type 1A (CMD1A) is a rare autosomal recessive disorder caused by mutations in the *LAMA2* gene. CMD1A is characterized by peripheral hypotonia and muscle weakness from the first months of life, cerebral white matter abnormalities, and elevated creatine phosphokinase (CPK) levels. We describe an 8-year-old girl from Colombia with clinical features compatible with CMD1A, severe scoliosis corrected with surgery, and feeding difficulty corrected with a gastrostomy. Whole-exome sequencing identified two heterozygous variants: a reported nonsense variant (*LAMA2* NM_000426.3:c.4198C>T) and a novel likely pathogenic variant (*LAMA2* NM_000426.3:c.9227_9243dup). This is the first genetically confirmed case of CMD1A in Colombia and the first report of the c.9227_9243dup variant causing CMD1A.

## Introduction

Congenital muscular dystrophy (CMD) is a general term for a heterogenous group of genetic disorders with overlapping phenotypic features such as progressive muscle degeneration and hypotonia caused by defects in skeletal muscle structural proteins (Amin et al., 2019). Congenital muscular dystrophy type 1A (CMD1A) is characterized by hypotonia and muscle weakness during the first months of life, delay in motor development, high levels of creatine phosphokinase (CPK), and alterations in the cerebral white matter (Liang et al., 2017; Smith et al., 2017). Possible complications include scoliosis, feeding difficulties, wheelchair dependence, and premature mortality, in which 30% of patients die within their first decade of life, commonly due to respiratory tract infections (Liang et al., 2017). Facial muscle weakness, ophthalmoparesis, and macroglossia are also features that may be present in these patients but often go beyond early childhood (Oliveira et al., 2018).

CMD1A prevalence is estimated at 1/30,000 and represents 30%–40% of all congenital muscular dystrophies, with some regional variation (Amin et al., 2019). CMD1A is not documented in Colombia except for the present case report and a previous case published in the city of Bogota diagnosed based on clinical, paraclinical, and imaging findings (Acosta Guío and Zarante Montoya, 2010). Therefore, epidemiologic data are unavailable in the country. This type of CMD is caused by mutations in the laminin subunit alpha 2 (*LAMA2*) gene in chromosome 6q22.33.

CMD diagnosis takes advantage of specialized tests such as the biopsy of affected muscle tissue to reveal characteristic changes in muscle fibers, such as the complete absence of staining or reduced/irregular staining of laminin-α2 by immunohistochemistry (Oliveira et al., 2018; Dimova & Kremensky, 2018; Hashemi-Gorji et al., 2018). Nevertheless, most CMD1A patients are too young to undergo a muscle biopsy (Liang et al., 2017). Magnetic resonance imaging (MRI) is also used to assess typical white matter changes in the brain, although these are detectable only after 6 months of age (Oliveira et al., 2018). Other tests that guide the diagnosis are elevated serum CPK concentrations and electromyography (Hashemi-Gorji et al., 2018). However, molecular genetic tests allow an accurate and timely diagnosis, determine the affected protein/process, and allow for establishing a prognosis depending on the severity of the mutation (Dimova and Kremensky, 2018).

*LAMA2* is a 9,684-nucleotide-long gene that expands through 65 exons and codifies a 3,118 amino acid protein, which, along with the β1 and γ1 chains, makes up laminin-211 (https://www.ncbi.nlm.nih.gov/gene/3908). Laminin-211 is an isoform that predominates in skeletal muscle, where it contributes to the union between muscle cells and the extracellular matrix (Smith et al., 2017). This compound protein is also expressed in the Schwann cells in the peripheral nervous system and the brain (Oliveira et al., 2018). During embryogenesis, laminin-211 plays a pivotal role in cellular migration and tissue arrangement (Amin et al., 2019). Thus, alterations in the expression of laminin-211 can affect skeletal muscle fiber integrity, leading to cell death and muscle degeneration (Smith et al., 2017). Mutations in *LAMA2* cause a wide phenotypic spectrum, which can range from an early-onset severe presentation to a later-onset milder type. Frameshift variants are the most common pathogenic variants in *LAMA2*, with a frequency of 82.5% (Amin et al., 2019). Here, we describe the case of a patient with clinical characteristics compatible with CMD1A and a novel deleterious frameshift variant in the *LAMA2* gene that had not been reported in the literature before.

## Case presentation

The patient, an eight-year-old girl from Colombia, was the third child of non-consanguineous healthy parents, born without complications via vaginal delivery. The parents stated that both siblings are healthy. They consulted the genetics area due to a history of peripheral hypotonia since the age of 2 months. At the age of 5 months, a simple cranial computed tomography (CT) scan was performed, confirming no alterations. At 1 year of age, brain MRI was performed, which reported white matter hyperintensity lesions on T2 and fluid-attenuated inversion recovery (FLAIR) adjacent to the occipital horns and bilateral hypodense signals in T1 associated with hypoxic-ischemic encephalopathy. Periventricular and occipital myelin disorders were present, but the criteria for leukodystrophy were not met.

At the age of 5 years, the patient underwent gastrostomy to provide enteral nutrition. Subsequently, another brain MRI was performed, which again showed an alteration of the deep white matter. An electromyogram (EMG) was performed, which showed an abnormal result with non-inflammatory myopathy in the upper and lower extremities.

**FIGURE 1:**
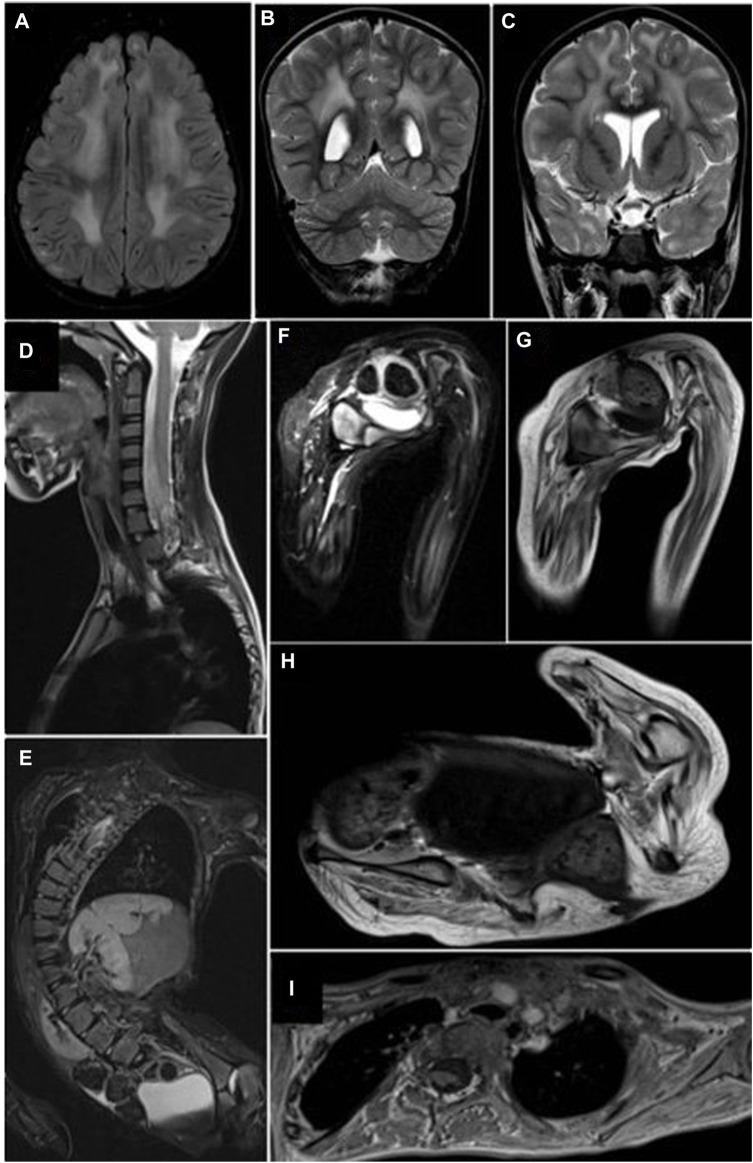
Cerebral and musculoskeletal system MRI. **(A–C)** Periventricular and subcortical white matter hyperintensities with a nonspecific demyelination pattern. FLAIR **(A)** and coronal T2 **(B, C)** sequences. **(D, E)** T2FS sequences. Thoracolumbar scoliosis secondary to muscular atrophy, without fusion defects or fragmentation of the vertebral bodies or compromise of the spinal canal. **(F, G)** Pelvis short tau inversion recovery (STIR) and T1 sequences. Diffuse and generalized muscular atrophy with Goutallier grade 3 fatty replacement without muscle edema. **(H,I)** T1 sequences of pelvic and shoulder girdle. Generalized decrease in muscle volume with Goutallier grade 3 fatty infiltration.

At the age of 6 years, an MRI of the musculoskeletal system was performed. Changes in the configuration of the pelvic ring were evidenced in the bony structures. Bilateral subluxation was observed in the coxofemoral joint, which correlates with dysplasia in the development of the acetabulum. The femurs presented marked hyperintensity on T1, suggesting probable osteopenic changes. In the femorotibial joint, slight hyperintensity of the growth plate was observed, which was also associated with osteopenic changes. The muscles of the pelvic girdle and thighs presented atrophic changes with loss of volume and marked fat replacement without showing foci of edema. In the spine, a severe right thoracolumbar scoliosis with a left cervicodorsal compensatory curve was observed. There was a rectification of the normal cervical lordosis with a flat back. In the thorax, atelectasis was observed in the upper and right posterior lobes, with a deformity of the thorax (Figure 1). There were no fusion defects or vertebral fragmentation. At the same age of 6 years, whole-exome sequencing (WES) was also performed. At the age of 7 years, the patient underwent surgery to correct scoliosis.

**FIGURE 2:**
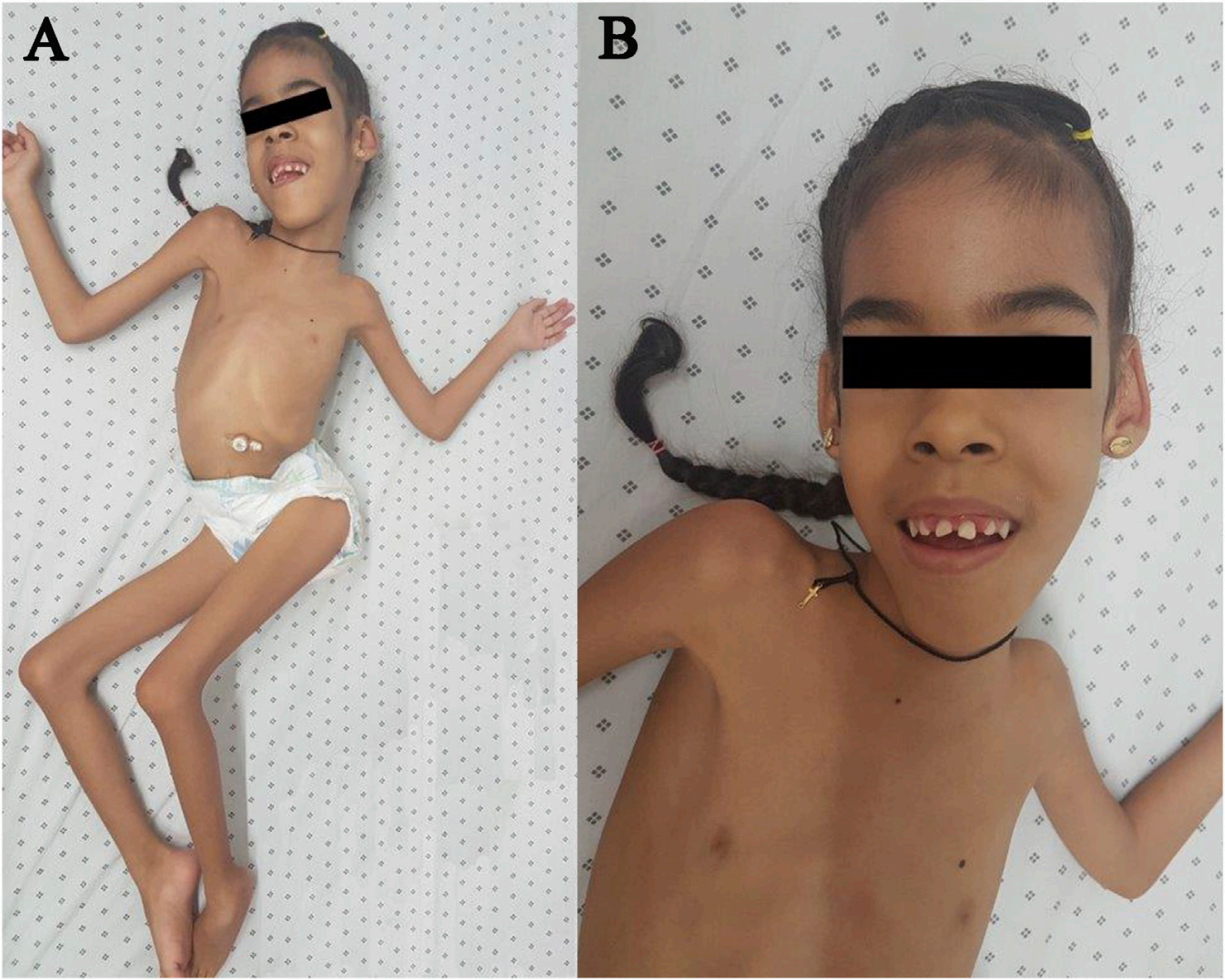
CMD1A Colombian patient. **(A)** Eight-year-old female patient with severe hypotonia, scoliosis, bilateral clubfoot, and hip dysplasia. **(B)** The patient presents elongatedmyopathic facies.

**FIGURE 3:**
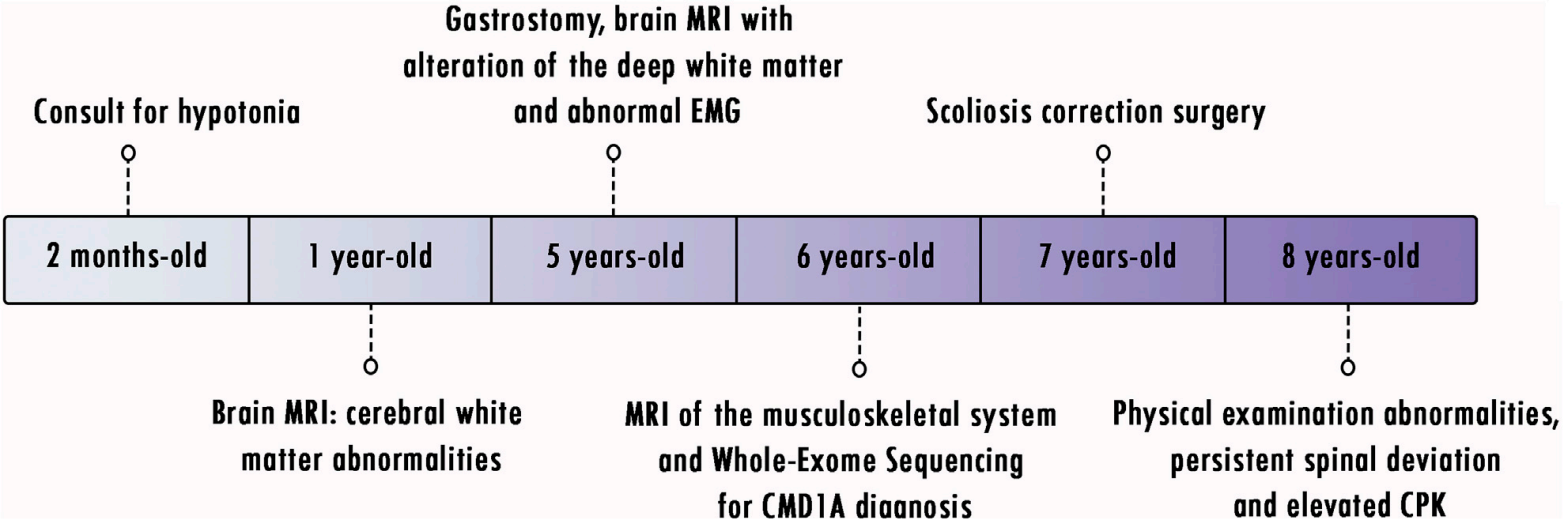
Timeline of clinical events.

At the age of 8 years, the patient weighed 16 kg, with a height of 118 cm, a body mass index (BMI) of 11.49, marked lumbar lordosis, spinal deviation, left pelvic obliquity, and instability of the left hip; knee flexion deformity persisted. Physical examination revealed a prominent high forehead, dolichocephaly, and retraction of the joints, mainly in the lower limbs (with difficulty in flexion and extension). The patient had severe hypotonia, scoliosis, bilateral clubfoot, and hip dysplasia (Figure 2). Spinal X-ray showed a reduction in the left hip in abduction. The chest X-ray showed the bars and screws that fixed the spine; however, an important right scoliotic curve could be seen. CPK levels in the patient remained elevated, with an average of 1,337–364 U/L. Clinical events are presented in Figure 3.

A trio-based WES on genomic DNA was performed at Centogene laboratories. Approximately 37 Mb of consensus coding sequences was enriched from genomic DNA fragments by >340,000 probes designed against the reference human genome (GRCh38) (Nextera Rapid Capture Exome kit, Illumina), and a library was generated and sequenced on the platform Illumina HiSeq 4000 sequencing system (Illumina, San Diego, CA, United States) to a depth of 100-130X reads. A proprietary bioinformatics approach was applied, including base annotation, first filtering of low-quality reads and possible artifacts, and variant annotation. A coverage of 99.7% of the target bases of the disease-causing gene was obtained for the patient and the mother and 99.9% for the father. An average coverage of 135.7X was obtained for the patient, 117.4 for the mother, and 117.8 for the father, considering that a base has sufficient coverage at 20X and an exon is considered completely covered if all coding bases plus three nucleotides of the intronic sequence on each side are covered at 20X or more. This clinical exome detected the pathogenic nonsense heterozygous variant *LAMA2*(NM_000426.3):c.4198C>T (p.Arg1400Ter) (Beytía et al., 2014) in the proband and the mother and the novel frameshift heterozygous variant *LAMA2*(NM_000426.3):c.9227_9243 dupTTGGCCTAACAACCAGT (p.Ile3082Leufs*3) in the proband and the father, which has not been reported in databases, such as Clinvar (RRID:SCR_006169), gnomAD, 1000 genomes (RRID:SCR_008801), and HGMD (RRID:SCR_001888), and is classified as a probably pathogenic variant according to the recommendations of the American College of Medical Genetics and Genomics (ACMG) (RRID:SCR_005769). This result confirmed the clinical diagnosis of CMD1A. No other clinically relevant variants were identified in this WES.

**FIGURE 4:**
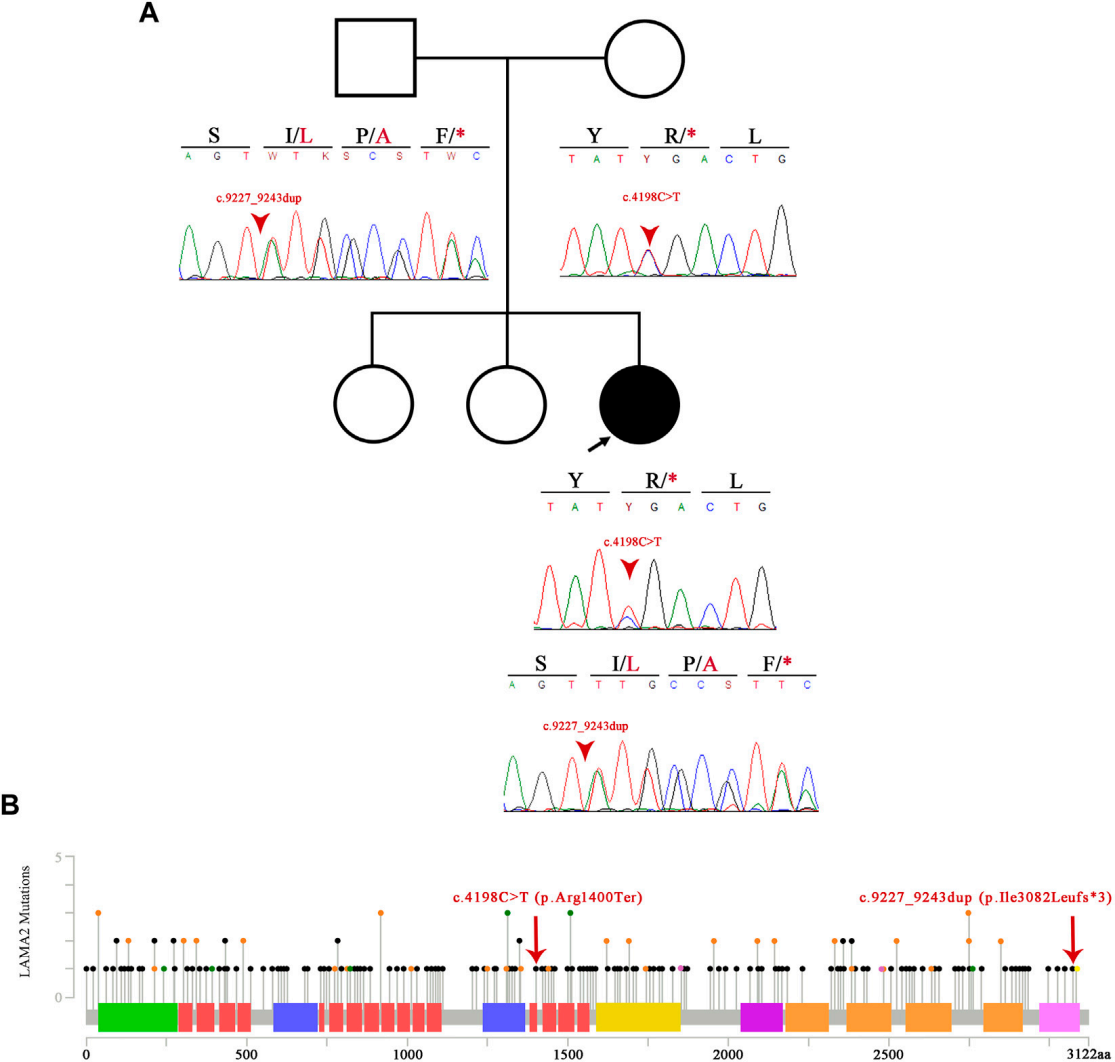
Pedigree and variant information. **(A)** Pedigree of the patient and Sanger sequencing electropherogram of the patient and her parents. The image shows the novel probably pathogenic variant p.Ile3082Leufs*3. **(B)** Image showing the spectrum of LAMA2 mutations alongside the two variants of our patient. To the right of the p.Ile3082Leufs*3 mutation, in yellow, lies the already published and truncating p.Arg3085* variant. The black dots showcase truncating mutations, the green dots represent missense variants, and the orange dots represent splicing variants. The laminin N-terminal domain is represented in green, laminin epidermal growth factor-like (EGF) domain in salmon, laminin B domain in purple, laminin domain I in yellow, laminin domain II in fuchsia, and laminin G domain in orange and pink.

In order to confirm these variants, blood samples were collected from both parents and the patient in 4 ml EDTA tubes, and DNA extraction was performed using the QIAamp DNA Mini Kit (QIAGEN, Germany) following the manufacturer’s protocol. Concentration and purity (260/280 and 260/230 ratios) of the nucleic acids were evaluated using a NanoDrop 2000 spectrophotometer (Thermo Fisher Scientific, United States). Polymerase chain reaction (PCR) primers for exons 29 and 65 of the *LAMA2* gene were designed by the authors: 5′- GCA​CTT​GCG​TTT​GTA​AGT​GAT​G-3′ and 5′- TTT​CAC​ACA​CAC​AGT​TGC​ATT​C-3′ for exon 29 and 5′- TCT​CAA​GCT​AAC​AGT​TGA​CTT​TG-3′ and 5′- ACT​CTT​CCT​GGG​GTT​ACA​C-3′ for exon 65. PCR was performed for both genes using the Promega (United States) GoTaq^®^ Flexi DNA Polymerase Kit in a final volume of 25 µL: 11.55 µL of water, 5 µL of 5X Green GoTaq^®^ Flexi Buffer, 4 µL of MgCl_2_ 25 mM, 1 µL of dNTPs 5 mM, 0.625 µL of each primer 10 µM, 0.2 µL of GoTaq^®^ Flexi DNA polymerase, and 100 ng of DNA. Both PCR amplifications were performed using the following thermocycling conditions: 95°C for 5 min, 35 cycles consisting of 95°C for 30 s, a specific annealing temperature of 61.5°C for 45 s, 72°C for 45 s, and a final extension at 72°C for 8 min. In order to ensure proper amplification, PCR products were separated by gel electrophoresis with agarose 1% at 100 V for 40 min, stained with ethidium bromide, and visualized using UV light. Subsequently, amplicons were purified with the QIAquick^®^ PCR Purification Kit (QIAGEN, Germany) and used for single-stranded Sanger sequencing using the BigDye Terminator v.3.1 (Applied Biosystems, Foster City, United States) following the manufacturer’s instructions. The Sanger sequencing products were then purified with the BigDye^®^ Xterminator™ Purification Kit (Applied Biosystems, Thermo Fisher Scientific, United States). Forward and reverse primers were used for the sequencing of both exons of the *LAMA2* gene using a 3500 Genetic Analyzer (Thermo Fisher Scientific, United States). Sequence data were analyzed using MEGA X software (Kumar et al., 2018) using the GenBank reference sequences of *LAMA2* (NG_008678.1). WES results were confirmed as the patient was compound heterozygous of the aforementioned variants, the father was heterozygous for the *LAMA2*(NM_000426.3):c.9227_9243dupTTGGCCTAACAACCAGT (p.Ile3082Leufs*3) variant, and the mother was heterozygous for the *LAMA2*(NM_000426.3):c.4198C>T (p.Arg1400Ter) variant (Figure 4).

## Discussion

CMD1A (MIM: 607855) is an autosomal recessive disorder caused by homozygous or compound heterozygous mutations in the *LAMA2* gene. Due to the phenotypic overlap and the clinical and genetic heterogeneity that neuromuscular disorders have, the diagnosis of CMD patients is achieved by DNA sequencing, especially next-generation sequencing (NGS), which is recommended by different groups as a diagnostic method to avoid the complications that a muscle biopsy can represent (Hashemi-Gorji et al., 2018;Saredi et al., 2019). The WES performed on our patient showed two compound heterozygous variants in the *LAMA2* gene. The *LAMA2*(NM_000426.3):c.4198C>T nonsense variant has previously been described as pathogenic in an individual affected with CMD1A (Beytía et al., 2014). This single-nucleotide variant creates a premature termination codon (PTC) in the protein (p.Arg1400Ter), which is expected to result in either a missing protein product or a truncated protein. This variant is found in the Genome Aggregation Database (gnomAD) with an allele frequency of 0.00000398, which is below the threshold for the recessive gene *LAMA2* (Dimova & Kremensky, 2018).

The novel frameshift variant *LAMA2*(NM_000426.3):c.9227_9243dup and its corresponding interpretation were submitted to ClinVar with Variation ID 2669005. This genetic variant alters the reading frame by duplicating a fragment of 17 base pairs, specifically in the isoleucine residue 3082, causing a stop codon 3 amino acids later (p.Ile3082Leufs*3). Loss-of-function (LOF) variants, including nonsense and frameshift variants, have an impact on the mRNA transcript and translated protein, often leading to disease phenotypes (Pagel et al., 2017). The literature describes that LOF variants for *LAMA2* have a pathogenic effect by producing truncated proteins or decreased mRNA due to nonsense-mediated transcript decay (Dimova & Kremensky, 2018). The most frequently reported genotypes in the *LAMA2* gene are variants that create PTCs, despite their location throughout the gene, such as the ones in our patient (Oliveira et al., 2018). When these types of variants are found in both alleles of the gene, they give rise to classic CMD1A, which is the early-onset, more severe form (Nguyen et al., 2019).

Milder and late-onset forms of the disease have generally been associated with missense variants, which are present in a smaller number of cases and generally correlate with a partial deficiency of laminin-α2 in immunohistochemical tests from muscle biopsies (Oliveira et al., 2018). Conversely, a complete absence of α2-laminin staining is associated with the presence of truncated mutations (Oliveira et al., 2008). However, this type of genetic variant exhibits a variable clinical phenotype, in which an individual homozygous for a pathogenic nonsense variant and laminin-α2 absent can achieve independent ambulation (Geranmayeh et al., 2010). The patient, in this case report, presented biallelic LOF variants consistent with a typical CMD1A early onset phenotype with severe symptoms starting at 2 months of age: delayed psychomotor development, regression in developmental milestones, compromised central nervous system, peripheral hypotonia and muscle weakness in all four limbs, altered brain MRI, and myopathic compromise on EMG.

Laminins are fundamental glycoproteins in the architecture and function of the basement membrane, where they bind to cell surface receptors through their laminin G-like (LG) domains (Yurchenco, 2015; Holmberg and Durbeej, 2013). Taking into account that the variant NM_000426.3:c.4198C>T (p.Arg1400Ter) is located in the LG5 domain, which, alongside the LG4 domain, presents binding sites for heparin and α-dystroglycan, it is possible that this mutation affects the union of laminin-α2 to molecules that bind the extracellular matrix and the sarcolemmal cytoskeleton, eventually affecting the stability of laminin-α2 (Geranmayeh et al., 2010; Holmberg & Durbeej, 2013). However, no functional analysis strategies are currently available for laminin-α2 variants, so the impact of these mutations remains unclear (Oliveira et al., 2018).

Muscle biopsy could not be obtained for functional studies. However, the amino acid affected by the stop codon of the c.9227_9243dup (p.Ile3082Leufs*3) variant, which is phenylalanine at position 3084, lies near another variant present in CMD1A patients (Figure 4). In patients with the c.9253C>T (p.Arg3085*) variant, immunohistochemical studies in muscle biopsy have shown a decrease in the amount of laminin-α2 (He et al., 2001), and histological studies have shown dystrophic features (O'Grady et al., 2016). Although the frameshift variant confirmed in this patient is not anticipated to result in nonsense-mediated decay, it is expected to lack the last 39 residues of the *LAMA2* protein. This evidence suggests that this is a clinically significant region of the protein. Available evidence indicates that the variant is pathogenic, but additional data are needed to prove that conclusively.

The patient in this case report presents typical clinical characteristics of CMD1A, such as peripheral hypotonia and muscle weakness, cerebral white matter abnormalities, elevated CPK levels during the first years of life (and their subsequent decline), joint contractures, severe scoliosis, hip dysplasia, elongated myopathic facies, feeding difficulty, and clubfoot, just as reported in the literature (Beytía et al., 2014). Other clinical features less reported in CMD1A are not expressed in our patient, such as cardiac problems, brain structural alterations, and ophthalmoparesis.

Our patient underwent a gastrostomy because CMD1A cases tend to develop feeding difficulties due to difficulty mastication and dysphagia, which can contribute to low weight and recurrent chest infections as a consequence of bronchial aspirations (Philpot et al., 1999). Our patient also had facial muscle weakness, resulting in elongated myopathic facies, such as the constant open mouth gesture, contributing to the eating and speaking difficulties. Scoliosis correction surgery was performed on the patient to avoid respiratory failure due to compression by the vertebrae, which would lead to the need for ventilatory assistance (Archer et al., 2016). All surgical procedures performed on the patient were tolerated, and there were no adverse or fortuitous events. In addition, since the COVID-19 pandemic began, the patient has not undergone new interventions, and she only continues with routine checkups.

## Limitations and strengths of the study

We thoroughly described the second case of CMD1A reported in Colombia and the first to be genetically confirmed through next-generation and Sanger sequencing. We provided a bioinformatic explanation of the pathogenic mutations and a comparison between the available literature regarding CMD1A and the clinical manifestations of our patient. Nevertheless, we were unable to provide functional evidence of the exact molecular mechanism underlying the pathogenesis of the CMD1A causing *LAMA2*:c.9227_9243dup variant, which needs to be further investigated and described. Finally, we were unable to perform functional studies on the muscle tissue of the patient due to laboratory capacity.

## Conclusion

This is the first genetically confirmed case of CMD1A in Colombia and the first report on the likely pathogenic variant *LAMA2* NM_000426.3:c.9227_9243dup as a probable cause of CMD1A. The clinical characteristics described in our patient correspond to those previously reported in the typical CMD1A early-onset phenotype, such as peripheral hypotonia and muscle weakness, cerebral white matter abnormalities, elevated CPK levels, joint contractures, severe scoliosis, hip dysplasia, elongated myopathic facies, feeding difficulty, and clubfoot. CMD1A features, such as cardiac problems, structural alterations of the brain, and ophthalmoparesis, are not presented in our patient. Functional and experimental data regarding the novel frameshift variant reported in this case report are required for future studies. Genetic diagnosis and counseling are emphasized because there are no prevalence or molecular diagnosis data regarding CMD1A in Colombia.

## Data Availability

The datasets for this article are not publicly available due to concerns regarding participant/patient anonymity. Requests to access the datasets should be directed to the corresponding author.

## Abbreviations

CMD, congenital muscular dystrophy; CMD1A, Congenital muscular dystrophy type 1A; CPK, creatine phosphokinase; *LAMA2*, laminin subunit alpha 2; CT, computed tomography; MRI, magnetic resonance imaging; EMG, electromyogram; WES, whole-exome sequencing; BMI, body mass index; PCR, polymerase chain reaction; NGS, next-generation sequencing; PTC, premature termination codon; gnomAD, genome aggregation database; LOF, loss-of-function; FLAIR, fluid-attenuated inversion recovery; STIR, Pelvis short tau inversion recovery; LG, laminin G-like; EGF, epidermal growth factor-like.
